# The interferon-inducible protein p202 promotes osteogenesis in mouse bone marrow stromal cells

**DOI:** 10.1042/BSR20171618

**Published:** 2018-06-27

**Authors:** Linlin Zhang, Chunhui Wang, Xianning Zhang, Haifang Li

**Affiliations:** College of Life Sciences, Shandong Agricultural University, Tai’an 271018, China

**Keywords:** Bone marrow stromal cells (BMSCs), Interferon-inducible protein p202, Inhibitor of differentiation (Id) proteins, Osteoblast differentiation, Runx2

## Abstract

In the present study, we explored the role of the interferon-inducible protein p202 in osteoblast differentiation of mouse bone marrow stromal cells (BMSCs). Both the mRNA and protein levels of p202 increased initially and decreased afterward in the course of BMSC osteogenesis. The intracellular distribution of this protein also changed in the differentiation process. p202 knockdown inhibited, while p202 overexpression enhanced, the osteoblast differentiation of BMSCs. This was identified by evaluation of expression of osteogenic markers, Alizarin Red S staining, and determination of alkaline phosphatase activity. Further study revealed that p202 disturbs the formation of Runx2/Ids complex and frees Runx2 to induce the differentiation process. The findings demonstrated that p202 plays a positive role in BMSC osteogenesis.

## Introduction

As a regulator that coordinates calcium and phosphate homeostasis, bone plays various physiological roles in vertebrates. It is an endocrine and hematopoietic organ as well, by secreting bone-derived hormones and hemocytes, which may regulate glucose homeostasis, kidney function, and even food intake [1,2]. Bone tissue keeps a dynamic balance between formation and resorption for life-long time, via the interplay of osteoblasts and osteoclasts. Osteoblasts are derived from mesenchymal stem cells and the tightly regulated process is controlled by a cascade of transcription factors [3,4]. For example, Runx2 (Runt-related transcription factor 2) is critical in the osteoblast-forming process, which regulates the expression of several bone-specific genes and controls the deposition of bone extracellular matrix [3,4]. However, the involved proteins in osteogenesis have not been elucidated completely so far. The osteogenic potential of bone marrow stromal cells (BMSCs) makes them selected tools for the investigation of the involved factors during osteoblast proliferation and differentiation [5].

The murine interferon-inducible p200 family is composed of more than ten homologous proteins including p202, p203, p204, p205, Aim2, Mndal et al. [6–8]. These family members contain either pyrin domain or HIN domains, or the both. They expressed in many tissues and act as important regulators in cell growth, apoptosis, immunomodulation, and tissue-specific differentiation [6–8]. p202, one of the most extensively studied p200 family members, functioned in cell-cycle regulation and differentiation via interaction with some transcription factors [9–11]. The important role of p202 in histogenesis has been demonstrated in studies targeting mesoderm-derived cells, such as myocytes, chondrocytes, osteoblasts, and adipocytes [9–11]. Although the involvement of p202 in osteogenesis has been previously reported [9], the underlying mechanisms need to be further investigated. For instance, Id (inhibitor of differentiation) proteins act as important regulators in cell growth and differentiation [12,13]. p204, another p200 family member, was found to be bound to Id proteins and overcame the inhibition of the Runx2 activity by the Id proteins, thus enhanced osteoblast differentiation in C2C12 cell lines and BMSCs [13]. Whether the interaction of p202 with Id proteins also exists during osteogenesis? This will be investigated in the present study.

In the present study, we first examined the temporal and spatial expression of p202 during the osteoblast differentiation of mouse BMSCs. Next, effects of p202 knockdown or overexpression on BMSC osteogenesis were determined, with regard to the alterations in transcription of osteogenic genes, mineralization, and alkaline phosphatase (ALP) activity. In addition, the underlying mechanisms were elucidated via co-immunoprecipitation (Co-IP) assay and luciferase reporter gene assay.

## Materials and methods

### Isolation and culture of mouse BMSCs

The present study was carried out in strict accordance with the recommendations in the Guide for the Care and Use of Laboratory Animals of Animal Care and Use Committee of Shandong province, China. The study was approved by the Animal Research Ethical Committee of Shandong province, China. BMSCs were acquired by accumulating the bone marrow of femurs and tibiae from Kunming mice (female, weighting 25 ± 5 g). Bone marrow cells suspended in DMEM were transferred into cell culture dish and cultured in an incubator with 5% CO_2_ at 37°C. Four days later, the nonadherent cells were removed. The adherent cells were cultured and passaged as previously described [5]. All the following experiments were performed using the third passage cells. The osteogenic degree of BMSCs was assessed in a complete culture medium added with osteogenic stimuli (OS), consisting of 0.01 μm dexamethasone, 50 μg/ml L-ascorbic acid, and 10 mm β-glycerophosphate. The fresh medium should be changed every 4 days.

### RNA extraction and quantitative real-time PCR (qRT-PCR)

Total cellular RNA was extracted using RNAiso plus reagent (Takara, China). RNA samples were reversely transcribed into cDNA using a reverse transcription kit (Takara). The mRNA levels of specific lineage markers were measured by qRT-PCR. The primer sequences for p202, GAPDH, Runx2, Col I (Collagen type I), and OCN (Osteocalcin) were listed in Table 1. qRT-PCR with SYBR Green was performed on a Bio-Rad real-time PCR system. Melt-curve analysis was conducted to verify that only one product was produced. The size of the DNA product was analyzed by electrophoresis in 1.2% (w/v) agarose gels. The mRNA levels of specific genes were calculated relative to the GAPDH levels using the 2^−∆∆*C*^_t_ method.

**Table 1:** Sequences of the primers or siRNA duplexes used in the present study

Name	Sequences (5′–3′)
p202	Forward: CAATGTCCAACCGTAACTT
	Reverse: ACTGTCATGGGTTTCTCATG
GAPDH	Forward: GACTTCAACAGCAACTCCCAC
	Reverse: TCCACCACCCTGTTGCTGTA
Runx2	Forward: CCGCACGACAACCGCACCAT
	Reverse: CGCTCCGGCCCACAAATCTC
Col I	Forward: CTGCCTGCTTCGTGTAAA
	Reverse: ACGTTCAGTTGGTCAAAGGTA
OCN	Forward: GAGCCCTTAGCCTTCCAT
	Reverse: GCGGTCTTCAAGCCATAC
siRNA-p202	Forward: GCUGGUUGAUGGAGAGUUU
	Reverse: AAACUCUCCAUCAACCAGC
siRNA-control	Forward: UUCUCCGAACGUGUCACGU
	Reverse: ACGUGACACGUUCGGAGAA
p202-fragment	Forward: CCC**AAGCTT**ATGTCCAACCGTAACTTAAGGTCATCTAC
	Reverse: CCG**CTCGAG**TCATTTTTCAGGCATGATGACCTCCAT
OCN-promoter	Forward: CCC**ACGCGT**ATCAAGCGGGCTCCTCAC
	Reverse: GGG**CTCGAG**ACCCTCCAGCGTCCAGTA

The restriction enzyme sites were labeled with bold.

### Western blot analysis

Total cell proteins, cytoplasmic, and nuclear fractions were collected respectively, according to the manufacturer’s instructions (Beyotime, China). Aliquots of protein from each sample were used to determine the expression patterns of p202 and Runx2, with GAPDH or Histone H3 as the internal control. The antibodies were from Abcam or Santa Cruz Biotechnology. Proteins were subjected to SDS-PAGE on a 12% polyacrylamide gel and transferred onto a PVDF membrane (Millipore, MA). After blocking with 5% nonfat milk in TBST (TBS containing 0.05% Tween-20), the membrane was incubated with the primary antibody and subsequently with the horseradish peroxidase-conjugated secondary antibody. The immunoreactive proteins were detected via chemiluminescence using an ECL kit (Beyotime, China). The images of Western blotting were quantified by Image-Quantity software (Bio-Rad, U.S.A.).

### Plasmid construction and transfection

Knockdown of p202 gene was performed using RNA interference technique. The specific nucleotide sequences of siRNA-control (nonspecific RNA duplex) and siRNA-p202 (siRNA specific to mouse p202 gene) were listed in Table 1. BMSCs were cultured in an antibiotic-free medium for more than 24 h. Subconfluent cells were exposed to Opti-MEM (Invitrogen) and transfected with either siRNA-p202 or siRNA-control (120 nM) using Lipofectamine 2000 (Invitrogen). Mock treatment (Lipofectamine 2000 only) was assigned in parallel. After 8 h, the medium was replaced with the induction medium containing OS (day 0).

To construct the recombinant p202 overexpression plasmid, the *Xho*I/*Hind*III fragments encoding the wild-type mouse p202 were cloned into pcDNA3.1(+), according to the manufacturer’s protocol. Briefly, the full length cDNA of p202 was amplified by a dynamic template PCR technique, using primers containing *Xho*I or *Hind*III restriction sites. The cDNA template and the pcDNA3.1(+) vector were separately cutted by *Xho*I and *Hind*III enzymes. The cutted cDNA fragments were ligated into the pcDNA3.1(+) vector (Promega, U.S.A.) to form pcDNA-p202. The correctness of the construct was confirmed by sequencing. BMSCs were treated as described above and transfected with either vector alone (pcDNA) or the p202-containing vector (pcDNA-p202) (400 ng/ml) employing Lipofectamine 2000.

### ALP activity assay

On day 4, treated cells were washed with PBS and lysed by two cycles of freezing and thaw. ALP activity in the cell layer was measured colorimetrically by monitoring the release of *p*-nitrophenol from *p*-nitrophenyl phosphate at 37°C using an ALP assay kit (Nanjing Jiancheng Biotech., China). Production of 1 mg *p*-nitrophenol at 37°C in 15 min was described as 1 unit (U). ALP activity was normalized to total protein determined using a BCA assay kit (Beyotime), which expressed as U/g protein.

### Alizarin Red S (ARS) staining

On day 8, ARS staining was performed to detect the mineralized nodules in treated cells, as previously described [14]. Briefly, the cells were fixed with 95% ethanol for 30 min at room temperature (RT). The fixed cells were washed with PBS and stained for 30 min with 40 mm ARS (pH 4.2) at RT with rotation. After washing with distilled water five times and rinsing with PBS for 15 min, whole well photographs were taken with a camera. Additionally, the bound dye was eluted with 10% cetylpyridinium chloride, and ARS in samples was quantified by measuring absorbance at 550 nm. Parallel wells served for DNA isolation by using a standard kit. The stained ARS was normalized to total DNA content and expressed as µmol ARS/µg total DNA.

### Co-IP assay

Co-IP assay was employed to test the binding of p202 or Runx2 to other proteins functioning in osteogenic differentiation*.* Treated cells were harvested and aliquots were incubated with the anti-p202 or anti-Runx2 antibodies (1 μg) at 4°C overnight. The cell extracts were removed after being incubated with protein A/G Plus Agarose beads. The proteins connected to the beads were released and examined by Western blotting with anti-p202, anti-Runx2, anti-Id1, anti-Id2, or anti-Id3 antibodies.

### Luciferase activity assay

To obtain luciferase reporter constructs, the appropriate DNA segments containing the promoter region of OCN were cloned into the pGL3-basic vector, which named as pGL3-OCNp. The primers with restriction enzyme sites were shown in Table 1. BMSCs were cotransfected with 400 ng/ml of pGL3-OCNp, pGL3-control, or pGL3-basic along with 20 ng/ml of pRL-TK respectively. Four days later, the treated cells were lysed in 100 µl of lysis buffer (Dual reporter assay system, Promega). The firefly luciferase activity was examined according to the protocols, and efficiencies were normalized to renilla luciferase activity directed by a cotransfected control plasmid pRL-TK.

### Statistical analysis

All experiments were conducted at least three times. Data were expressed as average ± SD. Origin software was used to assay the differences among samples, either by the one-way ANOVA or by two-sample Student’s *t-*test. *P*<0.05 was significant in statistical analysis.

## Results

### Expression profile of p202 during osteoblast differentiation of BMSCs

**Figure 1:**
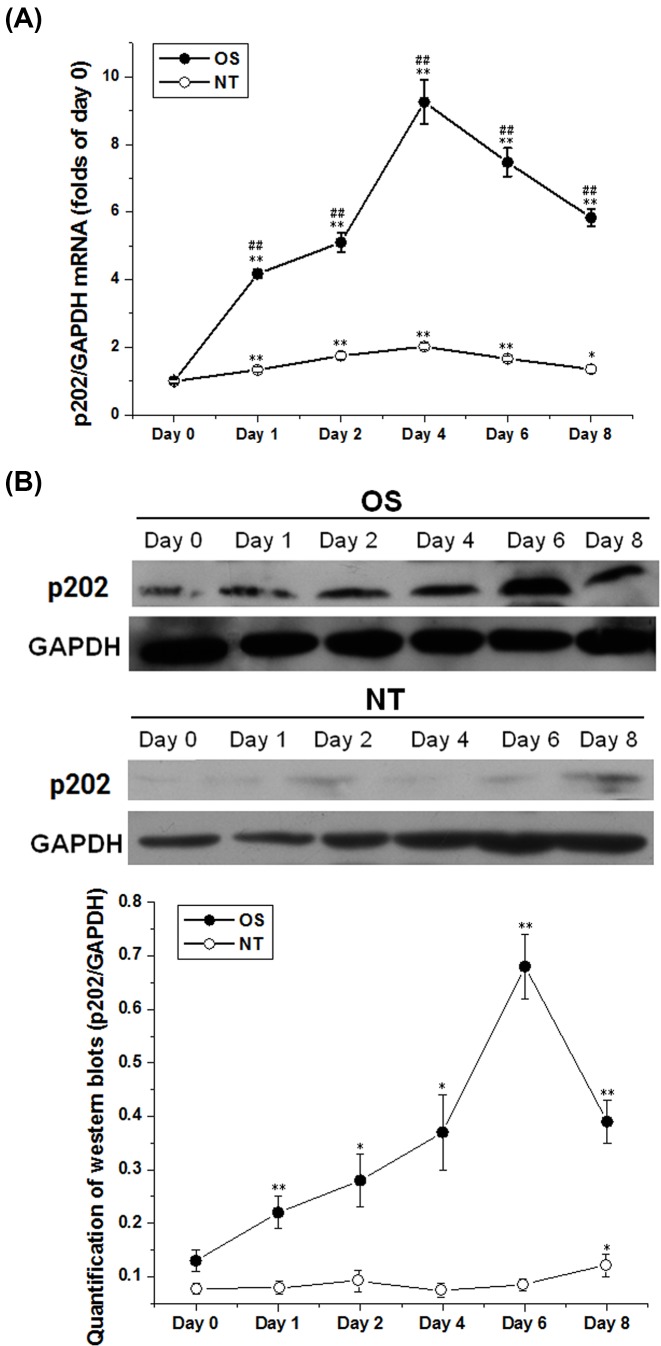
Time-course expression of p202 in BMSC osteogenesis BMSCs with or without OS induction were collected at day 0, 1, 2, 4, 6, and 8 respectively. Total RNA and protein were extracted from the cells, and qRT-PCR and Western blotting were performed respectively. (**A**) Temporal mRNA levels of p202 in BMSC osteogenesis assayed by qRT-PCR. The values were calibrated against day 0 control values that were set to “1.00”. Data were means ± SD of three separate experiments; *P <0.05, **P<0.01 vs. day 0; ^##^*P*<0.01 vs. NT (no treatment) at the corresponding time point. (**B**) Time-course expression of p202 protein in BMSC osteogenesis assayed by Western blotting. GAPDH was used as an internal control. Similar results were seen in another two experiments. The result was quantified and shown in a chart; *P<0.05, **P<0.01 vs. day 0.

The mRNA and protein levels of p202 during BMSC osteogenesis were determined by qRT-PCR and Western blotting respectively. As shown in Figure 1A, p202 mRNA was strongly increased in the osteoblast differentiation of BMSCs, which was peaked at day 4 (9.25-fold of day 0). However, the mRNA level of p202 showed a slight decline at day 6 and 8, which was 7.47- and 5.83-fold compared with day 0 respectively. Western blot analysis (Figure 1B) indicated that p202 protein elevated increasingly before day 6, while fell at day 8. The band density was increased by 0.69-, 1.15-, 1.85-, 4.23-, and 2.00-fold at days 1, 2, 4, 6, and 8 respectively, in contrast with that at day 0. In contrast, in treatment not supplementing with OS (NT), neither the mRNA nor the protein expression level was elevated significantly (Figure 1). This suggested that the alteration of the p202 expression was caused by the osteogenic induction of BMSCs.

### Subcellular distribution of p202 in osteoblast differentiation of BMSCs

**Figure 2:**
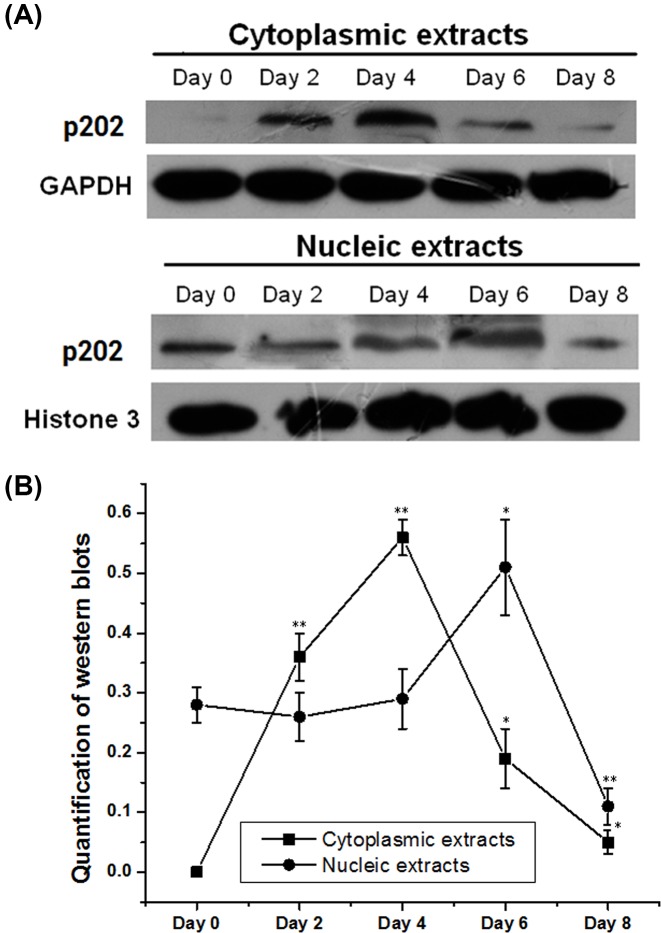
Subcellular localization of p202 at the indicated time points BMSCs with OS induction were collected at day 0, 2, 4, 6, and 8 respectively. Cytoplasmic and nucleic proteins were extracted from the cells respectively. Western blotting was conducted to see the p202 distribution in cytoplasmic and nucleic compartments during osteogenesis. (**A**) Intracellular detection of p202 at days 0, 2, 4, and 8 in cytoplasmic and nucleic compartments assayed by Western blotting. Similar results were seen in another two experiments. (**B**) The Western result was quantified and shown in a chart; *P<0.05, **P<0.01 vs*.* day 0.

The subcellular distribution of p202 was detected by immunoblotting using cytoplasmic and nuclear extracts at the indicated days (Figure 2). In the cytoplasm, p202 was barely detectable at day 0. It was induced sharply at day 2 and 4, while was reduced gradually at day 6 and 8. In the nuclear extracts, p202 could be observed at all days, with no significant variation among day 0, 2, and 4. However, there was an apparent elevation at day 6 (1.76-fold vs. day 4) and an evident reduction at day 8 (0.21-fold vs. day 6).

### Knockdown of p202 retards osteoblast differentiation

**Figure 3:**
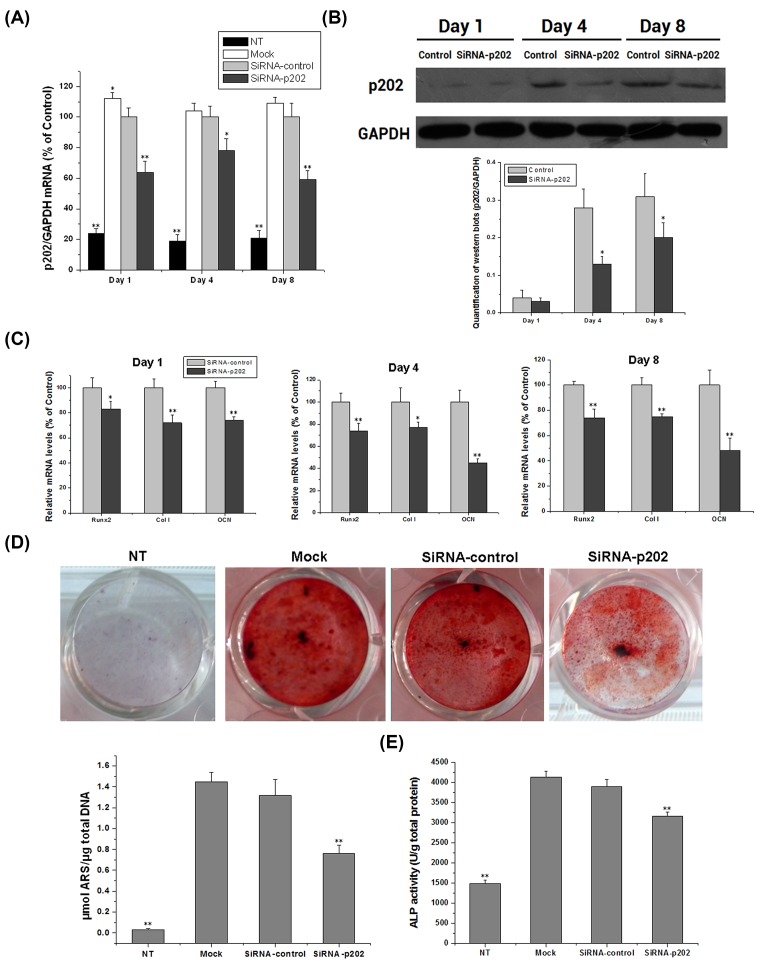
SiRNA against p202 mRNA impairs osteogenesis in BMSCs BMSCs transfected with siRNA-p202, siRNA-control, or mock were collected at day 1, 4, and 8 post OS induction. The cells were used to detect the mRNA and protein expression of p202 and osteogenic markers. On day 4, ALP activity in treated cells was examined. On day 8, ARS staining was perfomed to detect the mineral nodules in treated cells. (**A**) SiRNA-p202 efficiently inhibits the mRNA expression of p202. Data were expressed as means ± SD after normalization to values of control at each time point; *P<0.05, **P<0.01 vs. siRNA-control. (**B**) SiRNA-p202 reduces the p202 protein content. Similar results were seen in another two experiments. The result was quantified and shown in charts; *P<0.05 vs*.* siRNA-control. (**C**) The mRNA levels of osteogenic genes in siRNA-control- and siRNA-p202-transfected cells at days 1, 4, and 8. Data were expressed as means ± SD after normalization to values of siRNA-control; *P<0.05, **P<0.01 vs*.* siRNA-control. (**D**) The formed mineral nodules were identified by ARS-staining in treated cells on day 8. The stained ARS content in different cultures was expressed as µmol ARS/µg total DNA; **P<0.01 vs*.* siRNA-control. (**E**) ALP activity in treated cells on day 4, which was expressed as U/g total protein; **P<0.01 vs*.* siRNA-control.

To test whether p202 is required for osteoblast differentiation, siRNA-p202 was used to silence the expression of p202. BMSCs were transfected with siRNA-p202, siRNA-control, or mock. Results in Figure 3A,B showed that siRNA-p202 inhibited the expression of p202 effectively. SiRNA-p202 reduced its mRNA levels by 36, 22, and 41% at day 1, 4, and 8 respectively, comparing with siRNA-control. Similarly, siRNA-p202 exhibited significant suppression on the endogenous p202 protein at day 4 and 8 (54 and 35% inhibition respectively).

The transcription of genes typically induced during osteogenesis was down-regulated by siRNA-p202 (Figure 3C). For instance, OCN mRNA was restrained by 26, 55, and 52% at day 1, 4, and 8 respectively. In parallel, the mRNA levels of Runx2 and Col І were also inhibited by various percentages at the indicated days. After induction with OS for 8 days, ARS-staining showed that the mineral nodules were positive in mock, siRNA-control-treated, and siRNA-p202-treated cells (Figure 3D). However, siRNA-p202-treated cells showed reduced stained nodules (42% less ARS content vs. control). ALP activity was also significantly inhibited by siRNA-p202 treatment (19% inhibition vs. control) (Figure 3E). To a certain degree, p202 was required for BMSC osteogenesis.

### Overexpression of p202 stimulates osteoblast differentiation

**Figure 4:**
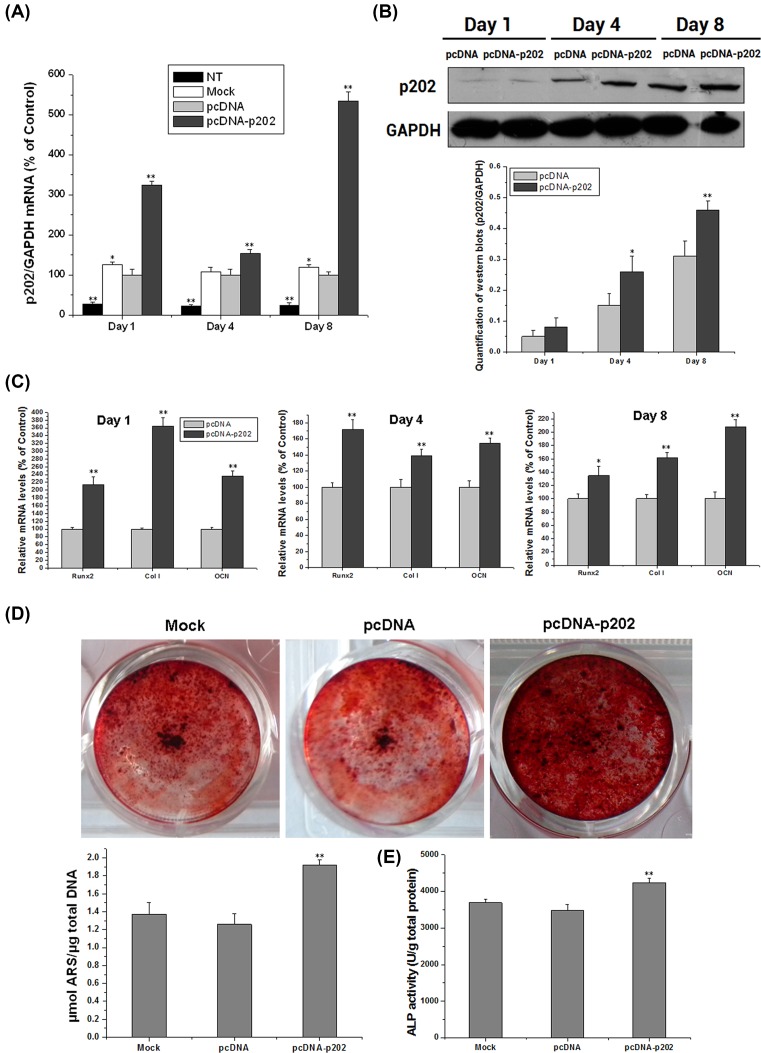
Overexpression of p202 by plasmid transfection enhances osteogenesis in BMSCs BMSCs transfected with pcDNA-p202, pcDNA, or mock were collected at day 1, 4, and 8 post OS induction. The cells were used to detect the mRNA and protein expression of p202 and osteogenic markers. On day 4, ALP activity in treated cells was examined. On day 8, ARS staining was perfomed to detect the mineral nodules in treated cells. (**A**) The mRNA levels of p202 during differentiation in NT, mock, pcDNA, and pcDNA-p202 treated cells. Data were expressed as means ± SD after normalization to values of pcDNA at each time point; *P<0.05, **P<0.01 vs. pcDNA. (**B**) The protein contents of p202 in pcDNA, and pcDNA-p202 transfected cells at days 1, 4, and 8. Similar result was seen in another two experiments. The result was quantified and shown in a chart; *P<0.05, **P<0.01 vs*.* pcDNA. (**C**) The mRNA levels of osteogenic genes in treated cells at days 1, 4, and 8. Data were expressed as means ± SD after normalization to values of pcDNA; *P<0.05, **P<0.01 vs*.* pcDNA. (**D**) The formed mineral nodules were identified by ARS staining in treated cells on day 8. The stained ARS content in different cultures was expressed as µmol ARS/µg total DNA; **P<0.01 vs*.* pcDNA. (**E**) ALP activity in treated cells on day 4, which was expressed as U/g total protein; **P<0.01 vs. pcDNA.

We also tested the impact of p202 overexpression on osteoblast differentiation. As shown in Figure 4A, with pcDNA-p202 treated, p202 mRNA was increased by 224, 54, and 435% at day 1, 4, and 8 respectively, compared with pcDNA-treated alone. pcDNA-p202 also increased the protein expression of p202 significantly at day 4 and 8, but not at day 1 (Figure 4B). qRT-PCR revealed that the mRNA expression of osteogenic genes was typically improved in pcDNA-202-treated cells. At the indicated days, the mRNA levels of Runx2, Col I, and OCN were all increased significantly by pcDNA-202 treatment, in contrast with pcDNA alone (Figure 4C). An obvious difference can be observed in the ARS-stained mineral nodules between pcDNA- and pcDNA-202-treated cells. pcDNA-202-treated cells showed 52% more ARS content vs. pcDNA treatment (Figure 4D). Moreover, pcDNA-202 treatment led to 21% elevation on the ALP activity, comparing with pcDNA treatment (Figure 4E).

### p202 knockdown or overexpression affects the association of p202/Runx2 with Id2

**Figure 5:**
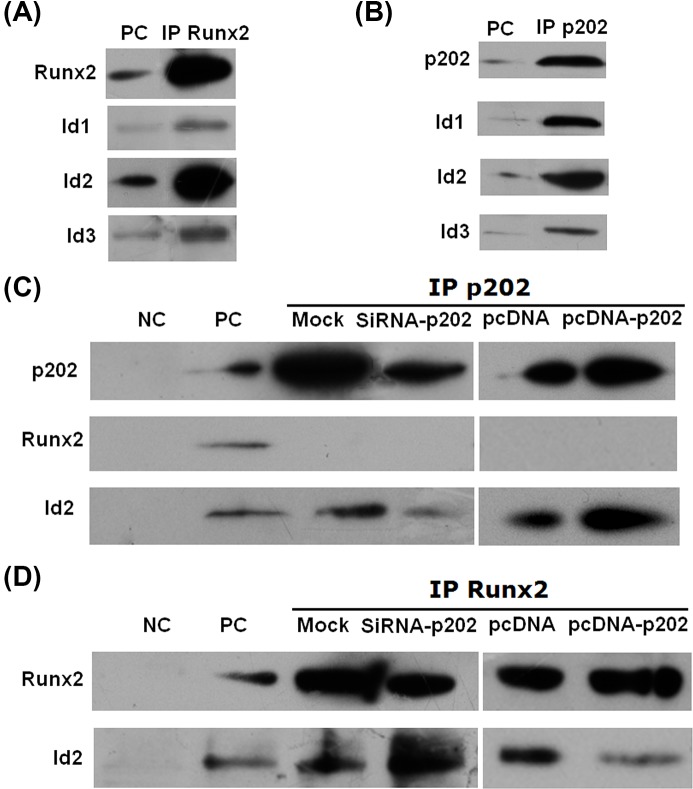
Interactions among p202, Runx2, and Id proteins determined by Co-IP assay BMSCs treated with OS, siRNA-p202, pcDNA-p202, pcDNA, or mock were collected at day 4. After immuneprecipitation with Runx2 or p202, the protein levels of Runx2, p202, or Ids were determined by using Western blot analysis. (**A**) Id proteins were detectable in Runx2-immuneprecipitated proteins at day 4. Extract from OS-treated cells without IP treatment was used as a positive control (PC). (**B**) Id proteins were found to be associated with p202 at day 4. (**C**) SiRNA-p202 inhibited, but pcDNA-p202 elevated, p202 and the p202-associated Id2 contents. Runx2 was not seen in p202-immuneprecipitated proteins. The anti-GAPDH antibody served as Control IgG (NC) to exclude nonspecific affiliations. (**D**) SiRNA-p202 reduced the Runx2 protein, while reversely increased the Runx2-associated Id2 content. However, pcDNA-p202 showed just opposite effect, in comparision with those in siRNA-p202 treatment.

Runx2 is a critical transcription factor in osteogenesis [3]. Id proteins are also involved in osteoblast differentiation [13]. To see whether direct association exists among p202, Runx2, and Id proteins, a series of Co-IP assay was performed at day 4 post induction. Figure 5A showed that Runx2 interacts with Id1, Id2, and Id3, which was consistent with previous reports [13]. We also found the association of p202 with Id proteins and Id2 was a major associated Id protein (Figure 5B). However, no interaction was seen between p202 and Runx2 (Figure 5C).

In order to further detect the role of Id proteins in the effect of p202 on osteogenesis, protein extracts from mock, siRNA-p202, pcDNA, or pcDNA-p202-treated cells were immunoprecipitated with anti-p202 antibody, and the complexes were detected with anti-p202 and anti-Id2 antibodies. Results indicated that p202 bound to Id2 in all the treated cells. SiRNA-p202 suppressed the p202 protein level significantly and also markedly reduced the interacted Id2 protein content (Figure 5C). However, pcDNA-p202 markedly increased the p202 protein and the associated Id2 (Figure 5C). Figure 5D showed that siRNA-p202 down-regulated the Runx2 protein, whereas up-regulated the Runx2-associated Id2 content. In contrast, pcDNA-p202 elevated the Runx2 level, but lowered the Runx2-interacted Id2 (Figure 5D). In view of the above findings, p202 knockdown appears to dramatically suppress the p202-bound Id2 level, thus frees more Id2 to interact with Runx2, although Runx2 is also inhibited in siRNA-p202-treated cells. Comparing with p202 inhibition, p202 overexpression exhibited the opposite result.

### p202 knockdown represses the transcription of OCN

Results in Figure 5 indicated that siRNA-p202 may interfere with the transcription activity of Runx2. Because OCN is a gene downstream Runx2, a luciferase reporter assay was performed using the promoter region of OCN in response to p202 silencing.

**Figure 6:**
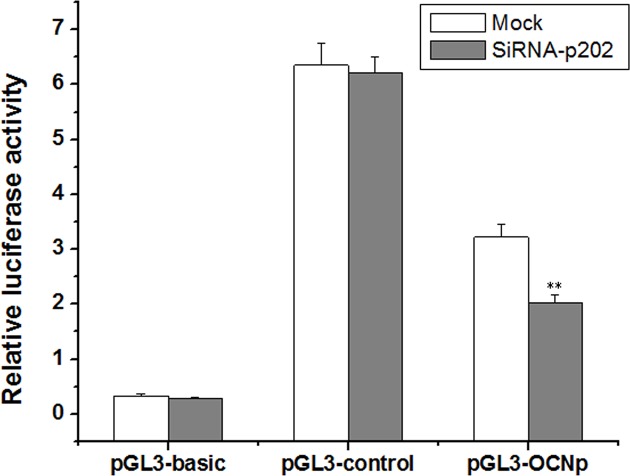
SiRNA-p202 suppresses the transcription of OCN, determined by luciferase reporter gene assay BMSCs were cotransfected with pGL3-OCNp, pGL3-control, or pGL3-basic along with pRL-TK respectively. On day 4, the treated cells were lysed and the firefly luciferase activity was examined. The relative luciferase activity was expressed as firefly luciferase activity/renilla luciferase activity. Values were means ± SD of three separate experiments; **P<0.01 vs*.* mock.

As shown in Figure 6, the relative activity of firefly luciferase to renilla luciferase in mock-treated cells was 3.22, while it was down-regulated into 2.03 in siRNA-p202-treated cells. It can be seen that siRNA-p202 significantly affects the transcription of OCN.

## Discussion

The interferon-inducible p200 family proteins are the gene products induced by interferons, which are important regulators in cell growth and differentiation [6]. p202 is a widely studied family member, which implicated in regulating hematopoietic cell proliferation and differentiation [15], hindering the growth of tumor [16], inhibiting the expression of MyoD protein during skeletal muscle differentiation [11], promoting osteoblast formation in C2C12 cell lines [9], and inducing adipocyte differentiation in mASCs [10]. In the previous study, p202 was found to be transactivated by Smad transcription factors and promoted osteoblast differentiation of C2C12 cells [9]. However, whether some critical proteins important for osteogenesis (such as Runx2, and Ids) are involved in the p202-regulated osteoblast differentiation has not been determined.

In the first, the expression pattern and subcellular location of p202 during BMSC osteogenesis were determined. Both the mRNA and protein levels were induced significantly after the start of the differentiation process, with day 4 and 6 showing maximal levels respectively (Figure 1). This result indicated that the gene encoding p202 is relatively an early response gene in osteoblast differentiation, and the protein variation lags behind the mRNA change. This pattern was similar but not identical with the time-course expression of p202 in osteoblast differentiation of C2C12 cells, in which p202 expression reached its highest level at day 2 following BMP-2 treatment [9]. The difference may be derived from the different cell types and the different induction system. p202 was identified as a nuclear protein that can shuttle between the nucleus and cytoplasm [17]. In the present study, p202 was almost exclusively located in the nucleus in undifferentiated cells. However, it also transferred to the cytoplasm in differentiating cells (Figure 2). This finding may support the physiological significance of the change in p202 localization during osteogenesis. Although the mechanism regulating p202 localization is not presently known, it is likely that it is critical for interactions with target proteins. Subsequent investigation showed this may be the case, since p202 specifically interacted with Id proteins (Figure 5B), which located both in nuclear and cytoplasm compartments [13].

To fully assess the role of p202 in BMSC osteogenesis, silencing and overexpression of this protein were achieved using a specific small interfering RNA and a recombinant plasmid respectively. Results demonstrated that p202 knockdown inhibited, while its overexpression enhanced, the osteoblast differentiation of BMSCs (Figures 3 and 4). The positive regulation of p202 for osteogenesis was supported by the mRNA changes of osteogenic genes, reduction or activation of ALP activity, and the altered ARS-stained mineral nodules. The implication of p202 in BMSC osteogenesis was in accordance with previous findings, in which p204, p202, and p205 positively modulated the osteoblast differentiation of C2C12 cells or BMSCs [9,18,19].

Finally, the mechanism by which p202 activates BMSC osteogenesis was determined. Runx2 is a critical transcription factor in osteogenesis. Previous study has detected the association of p204 with Runx2 [18]. However, in the present study, no interaction of p202 with Runx2 was seen (Figure 5C). p202 may lack an interacting structure with Runx2 protein. Id proteins are important suppressors in the differentiation of many cell types [13,20,21]. We found that Id proteins not only bound to Runx2, but also associated with p202 in the course of BMSC osteogenesis, and Id2 was a major associated family member (Figure 5A,B). It is possible that p202 disturbs the formation of Runx2/Ids complex and frees Runx2 to induce the differentiation process. Subsequent investigation demonstrated that this is the case. SiRNA-p202 dramatically lowered the p202-bound Id2, while enhanced the Runx2-associated Id2 content significantly. However, p202 overexpression increased the p202-bound Id2, but decreased the Runx2-interacted Id2 content (Figure 5C,D). Although siRNA-p202-treated cells showed a lower Runx2 content, its associated Id2 protein was reversely increased. This appears to be a result of the strong inhibition on p202 protein by siRNA-p202, which frees more Id2 to bind to Runx2. Further investigation showed that siRNA-p202 also reduced the transcription of OCN (Figure 6), which indicating the lowered transcription activity of Runx2, since OCN is a gene downstream Runx2 in osteogenesis [3]. A similar finding was reported previously, in which Runx2, Id proteins, and p204 formed a regulatory circuit and acted in concert to regulate osteoblast differentiation [13]. In view of the above findings, a scheduled program comes into being, in which p202 disturbs the formation of Runx2/Ids complex and frees Runx2 to induce the transcription of downstream genes, such as OCN, which further enhances the osteoblast differentiation of BMSCs. Collectively, the temporal and spatial expression patterns of p202, and the observed manners in which p202 affects the formation of osteoblasts from precursor cells, provide sufficient evidence that p202 functions as a positive regulator in osteoblast differentiation of BMSCs.

## Abbreviations

ALP: alkaline phosphatase
DMEM: Dulbecco’s modified Eagle’s medium
BMSC: bone marrow stromal cell
Id: inhibitor of differentiation
NT: no treatment
OCN: osteocalcin
OS: osteogenic stimuli
